# Metabolic activity in dormant conidia of *Aspergillus niger* and developmental changes during conidial outgrowth

**DOI:** 10.1016/j.fgb.2016.07.002

**Published:** 2016-09

**Authors:** Michaela Novodvorska, Malcolm Stratford, Martin J. Blythe, Raymond Wilson, Richard G. Beniston, David B. Archer

**Affiliations:** a School of Life Sciences, University of Nottingham, Nottingham NG7 2RD, UK; b Mologic Ltd, Bedford Technology Park, Thurleigh, Bedfordshire MK44 2YA, UK; c Deep Seq, Faculty of Medicine and Health Sciences, Queen’s Medical Centre, University of Nottingham, Nottingham NG7 2RD, UK; d Biological Mass Spectrometry Facility biOMICS, University of Sheffield, Brook Hill Road, Sheffield S3 7HF, UK

**Keywords:** *Aspergillus niger*, Conidial development, Manometry, RNAseq, Proteome, Sorbic acid

## Abstract

The early stages of development of *Aspergillus niger* conidia during outgrowth were explored by combining genome-wide gene expression analysis (RNAseq), proteomics, Warburg manometry and uptake studies. Resting conidia suspended in water were demonstrated for the first time to be metabolically active as low levels of oxygen uptake and the generation of carbon dioxide were detected, suggesting that low-level respiratory metabolism occurs in conidia for maintenance. Upon triggering of spore germination, generation of CO_2_ increased dramatically. For a short period, which coincided with mobilisation of the intracellular polyol, trehalose, there was no increase in uptake of O_2_ indicating that trehalose was metabolised by fermentation. Data from genome-wide mRNA profiling showed the presence of transcripts associated with fermentative and respiratory metabolism in resting conidia. Following triggering of conidial outgrowth, there was a clear switch to respiration after 25 min, confirmed by cyanide inhibition. No effect of SHAM, salicylhydroxamic acid, on respiration suggests electron flow via cytochrome *c* oxidase. Glucose entry into spores was not detectable before 1 h after triggering germination. The impact of sorbic acid on germination was examined and we showed that it inhibits glucose uptake. O_2_ uptake was also inhibited, delaying the onset of respiration and extending the period of fermentation. In conclusion, we show that conidia suspended in water are not completely dormant and that conidial outgrowth involves fermentative metabolism that precedes respiration.

## Highlights

• Resting conidia are demonstrated to be metabolically active.
• After triggering of conidial outgrowth fermentation occurs, followed by respiration.
• Sorbic acid inhibits O_2_ uptake and delays the onset of respiration.

## 1 Introduction

Conidia are asexual spores formed by moulds in enormous numbers, up to 10^9^ per cm^2^ of a mature colony. They are primarily formed for widespread distribution with the intention that at least a few conidia will arrive in suitable environments and will develop into mycelia. Conidia are designed to survive the physical traumas present during their distribution, particularly in their resistance to UV-light, extreme heat, and dehydration (drought) (Krijgsheld et al., 2013a). It has been shown that dry conidia of *Aspergillus fumigatus* and *Aspergillus nidulans* remain fully viable for at least a year (Osherov and May, 2001, Lamarre et al., 2008).

*Aspergillus niger* is a widely-distributed filamentous fungus, forming colonies with large clusters of black conidia. Conidia remain dormant in air or in water and tests on limited numbers of species have shown that the germination of conidia requires a triggering mechanism. Germination triggering for most fungi is related to sugars, amino acids and inorganic salts (Osherov and May, 2001). A combination of d-glucose and water is sufficient to initiate germination of *A. nidulans* conidia (Osherov and May, 2000) while specific pyranose sugars and l-amino acids have been shown to trigger germination in *A. niger* (Hayer et al., 2013, Hayer et al., 2014) irrespective of their potential metabolism. There was no evidence of conidial influx of the triggering compounds, and it is probable that the triggering structures outside the spores activate signalling pathways, such as cAMP/protein kinase A (PKA), RasA, or Ca^2+^/calmodulin (Osherov and May, 2000, Osherov and May, 2001, Krijgsheld et al., 2013b). The triggering/signalling results in isotropic conidial swelling and self-adhesion, followed by establishment of polarity, and the formation of a germ tube, and then mycelial growth (Momany and Taylor, 2000, Osherov and May, 2000). Transcriptome analysis of dormant conidia of *A. nidulans*, *A. fumigatus* and *A. niger* revealed high levels of pre-packaged mRNA in the conidia (Lamarre et al., 2008, Novodvorska et al., 2013, van Leeuwen et al., 2013a), including transcripts from genes involved in fermentation, gluconeogenesis, and the glyoxylate cycle.

The fermentation process is widely associated with yeasts, particularly the brewing yeast *Saccharomyces cerevisiae*, while most moulds appear to require abundant oxygen for growth (Miller and Golding, 1949). In reality, large numbers of yeast species do not ferment (Barnett et al., 2000) but generate energy using respiration mechanisms, and very few mould species grow anaerobically. Traditionally, fermentation was measured by collection of gas in inverted Durham tubes in broth cultures. Yeasts employing respiration also generate CO_2_ from the tricarboxylic acid cycle but this is exactly balanced by the uptake of oxygen by the mitochondria, for the cytochrome oxidase, resulting in no nett detection of gas. Fermenting yeasts absorb low levels of oxygen for use in biosynthetic reactions, particularly related to sterols and lipids (Fraenkel, 2011). The suggestion of fermentation in dormant mould conidia (Lamarre et al., 2008, Novodvorska et al., 2013, van Leeuwen et al., 2013a) is supported by the observations that no oxygen consumption was detected in *A. fumigatus* resting conidia, and oxygen uptake was detected after 3 h of germination (Taubitz et al., 2007). Respiration has been shown to be essential during the germination process (Brambl, 1975, Stade and Brambl, 1981). Anaerobic environments inhibit conidial germination although germination of conidia is inhibited at much lower oxygen levels that the inhibition of mould growth (Miller and Golding, 1949). The action of respiratory chain inhibitors, Rotenone and Antimycin A, allowed the swelling of conidia but not full germination to hyphae (Taubitz et al., 2007) and active mitochondria were detected in swollen spores and germlings. It was demonstrated that 1 mM cyanide inhibited respiration via the cytochrome *c* oxidase while the AOX (alternate oxidase) system, transferring electrons from ubiquinol to oxygen and bypassing the cytochrome *c* complex (Kirimura et al., 1999), was inhibited specifically by 1 mM SHAM. Fungal conidia provide a means of dissemination of the fungus in nature but also a source of infection of plants, animals and insects, and a means of contamination of food. The early stages of conidial development provide crucial events in pathogenesis and contamination and also provide handles for combatting disease and spoilage. For example, natamycin, an effective food preservative, specifically binds to ergosterol and inhibits germ tube formation (van Leeuwen et al., 2013b). In this study, we sought to improve the understanding of the early developmental events of conidial germination and took *A. niger* as our model system. Experiments were carried out to determine the metabolic activities in dormant conidia of *A. niger*, and during the early stages of the germination process, and to relate those metabolic events to a genome-wide analysis of gene expression and protein complement in the conidia. In addition, the action of the weak-acid food preservative, sorbic acid, was applied to study the mode of its action as full expression analysis of conidia exposed to sorbic acid has not previously been reported in moulds. Sorbic acid acted as a membrane active compound and inhibited the conidial germination process.

## 2 Material and methods

### 2.1 Media and growth conditions

*A. niger* strain N402 (Bos et al., 1988) was grown at 28 °C on *Aspergillus* complete medium at pH 4 (ACM) with composition as described previously (Novodvorska et al., 2013). Conidia were collected after 6–9 days by washing the agar slopes using 0.01% (w/v) Tween 80 at laboratory temperature, filtered through sterile synthetic wool and counted using a haemocytometer.

### 2.2 Warburg manometry

Respiration and fermentation in conidia were detected using constant-volume Warburg manometry. All experiments were carried out using single side-arm manometer flasks. 2 ml of conidia (10^9^/ml) re-suspended in water were pipetted into the flask and 0.4 ml water or 20% KOH in the centre well. Growth inhibitors and/growth media were put in single arm and then added to conidia at the appropriate time. Two control flasks contained water only. The spore suspension flask was warmed up for 10 min before measurements took place. Each experiment was performed in both technical and biological duplicates at 28 °C.

### 2.3 Trehalose assay

The trehalose assay was done in duplicates using cytosolic extracts prepared from dormant and germinating conidia over a 4 h period (0 h, 0.5 h, 1 h, 2 h and 4 h) ±1 mM sorbic acid. 10^9^ conidia were collected by centrifugation, washed with sterile water and re-suspended in 1 ml 0.25 M Na_2_CO_3_ and 0.5 ml glass beads and disintegrated in a Sartorius dismembrator for 4 min at 2000 rpm. Supernatants were used to assay for trehalose content using a commercially available kit according to the supplier’s instructions (Megazyme International, Ireland Ltd). The amount of trehalose was expressed in pg/spore.

### 2.4 Protein extraction, LC-MS/MS and data analysis

Conidia germinated in liquid ACM media ±1 mM sorbic acid for 1 and 5 h at 28 °C, were collected, washed in 50 mM of Tris-HCl pH 7.5 and snap frozen in liquid nitrogen. Pellets from all 4 time points (T1, T1SA, T5, T5SA) with 0.5 ml glass beads were separately disintegrated in a Sartorius dismembrator for 4 min at 2000 rpm. Soluble protein fractions were extracted by adding 1 ml of TRIS buffer pH 7.5, vortexed for 1 min, boiled for 5 min at 95 °C and supernatant was transferred into 2 ml collection tubes. Insoluble protein fractions were extracted by adding 1 ml of protein extraction buffer (4% w/v SDS, 100 mM Tris buffer pH 7.5, 100 mM DTT) to the pellet and treated the same way. Soluble and insoluble protein fractions from the same time point were combined and subjected to 10 min trichloroacetic acid precipitation on ice, collected by centrifugation and washed twice with ice-cold acetone. The protein pellet was then air-dried and dissolved in 50 mM TRIS buffer pH 7.5. Each sample was prepared in duplicate. Proteins in 35–70 μg of total protein extract were separated on 1D SDS-PAGE gels. After destaining, gels were cut to 1 mm pieces and the proteins were reduced, alkylated and digested by trypsin. Peptides were then collected by rounds of incubation with 100% acetonitrile, then 0.5% (w/v) formic acid at 37 °C for 15 min before being vacuum-dried and subsequently being solubilized in solution (0.1% formic acid, 3% acetonitrile). 50% of the material was injected, using a Dionex Ultimate 3000 HPLC, onto a PepMap100 C18 2 cm × 75 μm I.D. trap column (ThermoFisher Scientific) at 5 μl min^−1^ in 0.1% (w/v) formic acid, 2% (w/v) acetonitrile at 35 °C in the column oven, 6 °C in the autosampler. Components in the sample were separated, over a 2 h HPLC run (containing a 70 min separation gradient) using mass spectrometry (van Munster et al., 2014). The resulting spectra were searched using SequestHT (ThermoFisher Scientific) against a custom *A. niger* 588.13 database (http://www.aspergillusgenome.org/download/sequence/A_niger_CBS_513_88/current/), and a decoy database, within the Proteome Discoverer 1.4 software package (ThermoFisher Scientific). Full trypsin enzymatic specificity was required with up to 2 missed cleavages permitted. Carbamidomethylation of cysteine was specified as a fixed modification (+0.57.021 Da), with oxidation of methionine (+0.15.995 Da) and N-terminal acetylation (+42.011 Da) were specified as variable modifications. A mass tolerance of 10 ppm was used for precursors and 0.6 Da for fragment ions. For the protein to be reported as present a filter was applied requiring a minimum of two peptides of medium (⩾95%) confidence to be detected.

### 2.5 RNA extraction, RNA sequencing and data analysis

Dormant and conidia germinated ±1 mM sorbic acid for 1 h at 28 °C were collected and snap frozen in liquid nitrogen. Pellets from one timepoint/condition were combined and total RNA was extracted as described in Novodvorska et al. (2013). Two biological replicates were done for each timepoint/condition. RNA-seqencing was performed at the Next Generation Sequencing Facility (Queen’s Medical Centre, University of Nottingham, UK). 5 μg of total RNA was depleted of ribosomal RNA using the Ribominus Eukaryotic kit (Invitrogen). SOLiD whole transcriptome libraries were prepared, quantified and purified as described in Novodvorska et al. (2013). Sequencing was performed on a SOLiD 5500xl ABi sequencer according to the manufacturer’s instructions to generate 75 bp reads in colour space. Reads from each sequencing library were mapped to the annotated genome assembly of the *A. niger* CBS 588.13 (AspGD version s01_m06_r10) using the LifeScope 2.5.1 Whole Transcriptome Pipeline (LifeTechnologies). Reads were initially quality checked and then subsequently filtered against the sequences of library adaptors and *A. niger* rRNA. LifeScope then mapped the reads to the sequence of the entire genome as well as a library of all exon junctions, derived from the genome annotation information, thereby accounting for spliced reads. The primary read alignment position of each mapped read was recorded in BAM format for further downstream analysis. Read counts per gene were calculated from each sample BAM file using HTseq-count with read alignments with a minimum mapping quality score of 20 (MAPQ20) (PMID: 25260700). Normalised gene expression values (RPKM) (Reads Per Kilobase of gene model per Million mapped reads) (Mortazavi et al., 2008) were also calculated using a custom Perl script. Differential gene expression analysis was achieved using the R package DEseq using the read counts per gene for each sample according to replicate grouping. Significantly differentially expressed genes were determined according to an adjusted p-value of 0.05. Data files were submitted to the Gene Expression Omnibus, under accession number GSE79745.

### 2.6 Gene ontology enrichment analysis

FetGOat database http://www.broadinstitute.org/fetgoat/ (Nitsche et al., 2011) used for Gene Ontology (GO) enrichment analysis. Conditions were selected as follows: *A. niger* CBS513.88 strain, FRD q-value of 0.05 and Benjamini and Hochberg multiple testing corrections. For analysis were selected differentially expressed genes with increased or decreased transcript levels with p-value ⩽ 0.05 (DEseq) and also proteins detected in both proteome replicates from each time point/condition were analyzed.

### 2.7 Uptake of radioactive glucose

20 ml vials with 15 ml ACM containing 55 mM d-glucose and ±1 mM sorbic acid and 10 μCi d-[U-^14^C]glucose (Perkin-Elmer) and 3 × 10^7^/ml conidia were kept at 28 °C during the experiment with regular stirring. Every ten minutes 1 ml samples were taken and filtered through 25 mm cellulose nitrate filters (0.45-μm pore size) that had been prewashed with 5 ml of 55 mM d-glucose ACM media (with no ^14^C). Filters were then washed again 3 times using the same media and put into 5 ml ScintiSafe 3 liquid scintillation cocktail (Fisher Scientific, United Kingdom). Radioactive emissions were counted using a Packard Tri-Carb 2100 TR liquid scintillation analyzer and dpm values converted to uptake from d-[^14^C]glucose (nMol/10^7^ conidia).

## 3 Results

### 3.1 Resting conidia

**Fig. 1:**
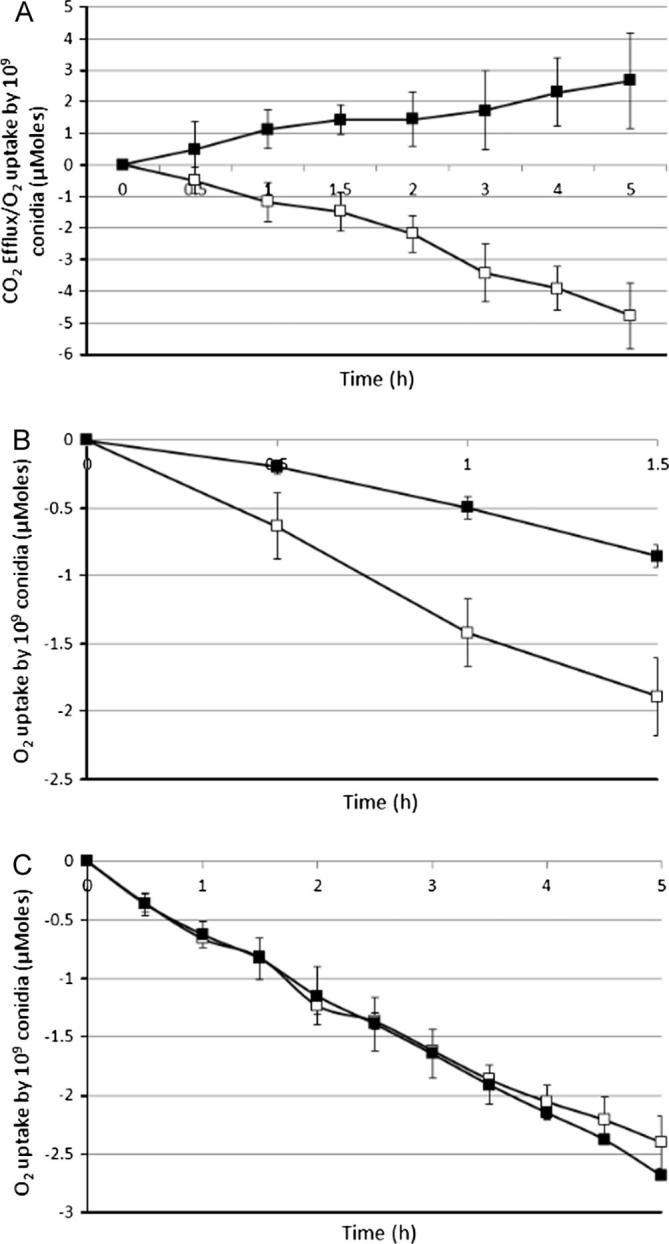
O_2_ uptake and CO_2_ efflux by dormant conidia in water at 28 °C. The means and standard deviations of duplicate samples are shown. (A) O_2_ uptake (open squares) and CO_2_ efflux (closed squares) over the period of 5 h. (B) O_2_ uptake in the presence (closed squares) and absence (open squares) of cyanide over the period of 1.5 h. (C) O_2_ uptake in the presence (closed squares) and absence (open squares) of SHAM over the period of 5 h.

Production of spores is induced by starvation or in stress conditions and is a prerequisite for survival of the fungus in harsh environments. Conidia can survive in a resting stage for long periods (Lamarre et al., 2008) and it is believed that they are metabolically inactive (Taubitz et al., 2007). We used manometry to explore whether there is metabolic activity in dormant conidia. Resting conidia, suspended in water, were shown not to be metabolically inactive as there were low levels of O_2_ uptake and generation of CO_2_. 10^9^ conidia took up 4.8 μmol of O_2_ and produced 2.7 μmol of CO_2_ over 5 h (Fig. 1A). O_2_ uptake was consistently greater than the CO_2_ efflux. The amount of evolved CO_2_ was never higher than (and not equal to) the amount of O_2_ taken up and, therefore, conidia performed respiratory metabolism over this period (see below for further interpretation of the data). In contrast, heat-killed conidia (75 °C, 15 min) showed no metabolic activity (no uptake of O_2_ or evolution of CO_2_; data not shown). Fermentation would be expected to produce much higher levels of CO_2_ efflux than O_2_ input while respiration would be expected to equally match CO_2_ efflux and O_2_ uptake. Using the data from 8 experimental runs, O_2_ uptake was 0.96 ± 0.35 μmol/h/10^9^ conidia while CO_2_ efflux was 0.60 ± 0.29 μmol/h/10^9^ conidia. The ratio therefore showed that O_2_ uptake was constantly 38.4% higher than the CO_2_ efflux. Cyanide, an inhibitor of the electron transport chain, specifically inhibiting cytochrome *c* oxidase, inhibited O_2_ uptake in dormant conidia by approximately 50% but had no impact on CO_2_ production (Fig. 1B). The inhibitor of alternative oxidase, SHAM, did not have any effect on oxygen uptake, nor on CO_2_ production. The presence of *aox1* (An11g04810) transcripts was detected in the transcriptome of resting conidia (T0), although AOX activity wasn’t detected (Fig. 1C). Additionally, detailed analysis of the T0 transcriptome showed the presence of transcripts of genes associated with oxidative phosphorylation (Supplementary file 1).

### 3.2 Conidial outgrowth

In order to study cellular responses during germination we performed a proteomic study at the first hour (T1) of conidial germination and compared the data with the transcriptome data. Two replicates from each time point were carried out and only proteins detected in both replicates were used in downstream analysis (Supplementary file 2). There were 672 proteins detected at T1. A total of 2487 genes increased their transcript levels and 2412 genes decreased their transcript levels during the first hour of germination. Transcriptome data showed that 5248 genes had transcript present with RPKM ⩽ 1. Out of them, 524 genes had their proteins detected. 140 genes (RPKM ⩾ 1) had proteins detected and represented mainly down-regulated genes. 8 genes with detected proteins had no transcripts present. GO enrichment analysis showed that carbohydrate metabolism, oxidative phosphorylation and translation represented the main categories of proteins. This correlated well with the transcriptome data showing respiration, translation, replication and transcription being the most represented groups from upregulated genes. Supplementary files 3 and 4 show the terminal node hierarchical GO terms in Biological Processes that were detected in the T1 proteome and transcriptome. We then focused on detailed analysis of specific genes related to energy metabolism and nutrient sensing.

**Fig. 2:**
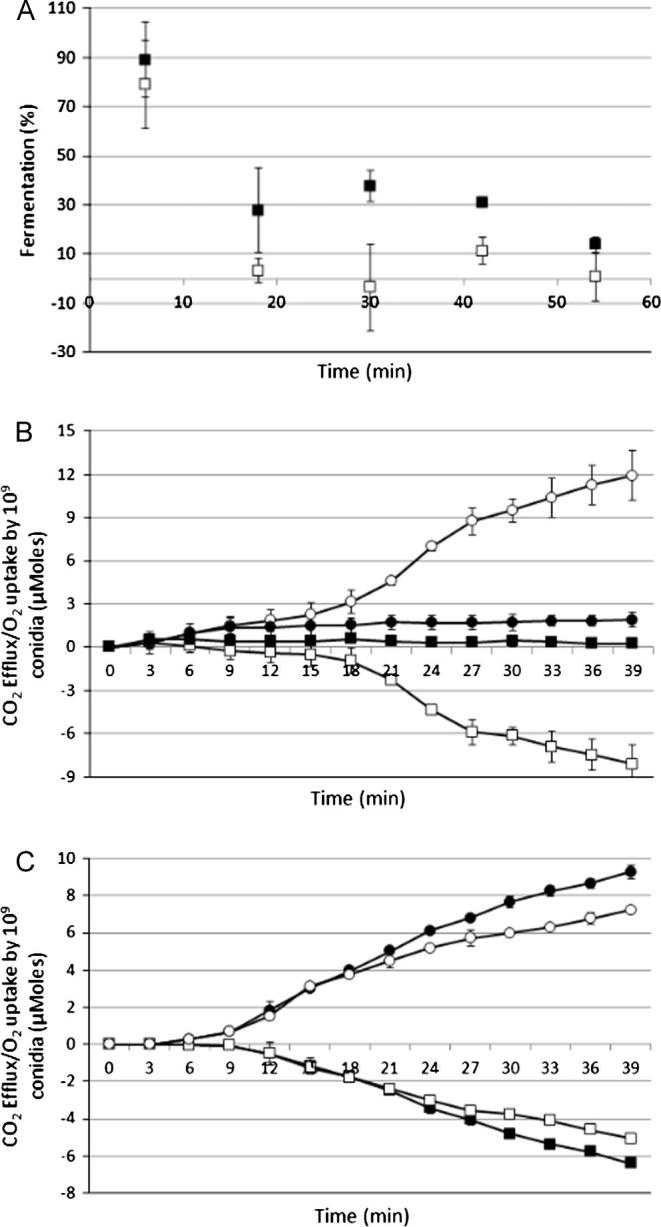
O_2_ uptake and CO_2_ efflux by 10^9^ conidia at 28 °C. The means and standard deviations of duplicate samples are shown. (A) Percentage of total CO_2_ efflux from fermentation in the presence (closed squares) and absence (open squares) of sorbic acid over the period of 54 min of germination. (B) O_2_ uptake (open squares) and CO_2_ efflux (open circles) by 10^9^ conidia without and with cyanide (closed squares, closed circles) over the first 39 min of germination. (C) O_2_ uptake (open squares) and CO_2_ efflux (open circles) by 10^9^ conidia without and with SHAM (closed squares, closed circles) over the first 39 min of germination.

**Fig. 3:**
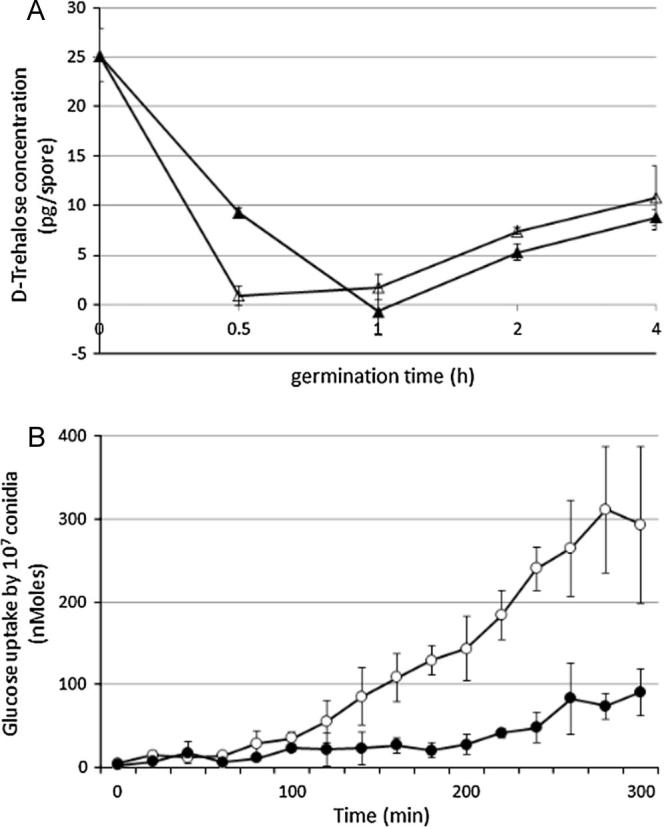
(A) d-Trehalose levels in conidia over 4 h of germination at 28 °C in the absence (open triangles) and presence (closed triangles) of sorbic acid. The means and standard deviations of duplicate samples are shown. (B) Uptake of d-[U-^14^C]glucose over the period of 5 h by 10^7^ conidia at 28 °C in the presence (closed circles) and absence (open circles) of sorbic acid. The means and standard deviations of duplicate samples are shown.

To produce a more comprehensive understanding of early development of *A. niger* conidia we assayed energy metabolism during germination using manometry. Results showed that excess CO_2_ was produced immediately upon germination triggering and O_2_ uptake was initiated after 12 min (Fig. 2A). Over the first 12 min of germination, high levels of CO_2_ were formed while the O_2_ uptake remained low, being consistent with the ratios expected during fermentation. After 12 min the O_2_ uptake progressively increased, reaching an equal level of CO_2_ output at 25 min. The ratio of CO_2_ output to O_2_ input was consistent with fermentation being replaced by respiration between 12 and 25 min. The effects of cyanide and SHAM were tested during conidial outgrowth in order to find out whether electrons are transferred to oxygen via cytochrome *c* or by alternative oxidase. 1 mM cyanide stopped uptake of O_2_ within 3 min and it had negligible effect on CO_2_ production over the first 12 min (Fig. 2B). There was no detectable uptake of O_2_ in the presence of cyanide. When cyanide was added 1 h after the growth was initiated, corresponding to full respiration, O_2_ uptake stopped immediately. Transcripts from the *aox1* gene (An11g04810) were present at T1 and we therefore tested if alternative oxidase was active, by using its inhibitor, SHAM. It had no effect over the first 20 min but afterwards SHAM appeared to marginally increase CO_2_ efflux and O_2_ uptake (Fig. 2C). This time also coincided with the mobilisation of intracellular trehalose that was most likely metabolised by fermentation (Fig. 3A). Transcripts of *treB* (An01g09290), encoding neutral trehalase, that is responsible for trehalose catabolism, were detected in the T1 transcriptome and TreB protein was detected in the T1 proteome.

We used radiolabelled d-[U-^14^C]glucose to follow the timing of entry of glucose into germinating conidia in complete medium. We discovered that glucose uptake was detectable 60 min after triggering outgrowth (Fig. 3B) and the rate of uptake increased exponentially afterwards. Approximately 300 nmol of glucose were taken up over the period of 5 h by 10^7^ conidia. Out of 72 genes encoding putative glucose/hexose transporters (Pel et al., 2007) transcription of 5 genes (An02g03540, An15g03940, An05g01290, An16g08940, An02g01270) was highly upregulated at T1. The most upregulated was the homolog of *A. nidulans mstE*, (An02g03540) encoding a low affinity glucose transporter. Transcription of *mstA* encoding a high affinity glucose transporter increased with germination time (unpublished results) and therefore might be necessary at later stages of germination or growth. G-protein coupled receptors (GPCRs) represent the largest group of transmembrane receptors with a diverse range of stimuli including sugars and amino acids (Maller, 2003). GPCRs initiate signalling cascades via heterotrimeric G-proteins. Nutrient detection by GPCRs results in the exchange of GTP for GDP on the Gα subunit leading to dissociation from Gβγ subunits (Xue et al., 2008). Regulators of G-protein signalling (RGSs) balance intensity and continuation of the G-protein signal (Yu, 2006). There are 12 genes in the *A. niger* genome encoding putative GPCRs and 5 of them (An02g01560, An09g06510, An08g04110, An07g09280, An01g00400) were transcribed in early germination (T1). 2 genes encoding Gα subunits (An08g06130 *fadA*, An02g08000 *ganA*) and 1 Gβ (An18g02090, *sfaD*) were also detected in the T1 transcriptome together with RGS proteins (An02g03160 *flbA* and An18g06110 *rgsA*). 5 genes encoding serine/threonine phosphatases, essential for glucose sensing and germination on glucose as sole carbon source regulating carbon source dependent germination, were characterized in *A. nidulans* (De Assis et al., 2015). An01g03700, An01g14160, An02g06630, An07g07110, An18g05890 homologs of *A. nidulans psrA*, *ptcD*, *ptpB*, *mgsA*, *ptcE* respectively, had their transcript detected at both time points (T0, T1). PtcE protein was also detected in T1 (Table 1).

### 3.3 Impact of sorbic acid on conidial outgrowth

In order to investigate how conidia respond to sorbic acid at the molecular level, we studied its impact on the transcriptome and proteome on outgrowing conidia and compared those data (e.g. T0–T1SA) with the non-sorbic acid data set (e.g. T0–T1). Supplementary file 1 contains RPKM values and differential gene expression analysis. A total of 1721 genes increased their transcript level and 1767 genes decreased their transcript levels at the first hour of conidial germination as a consequence of sorbic acid treatment. GO enrichment analysis using the list of differentially expressed genes (T1-T1SA) showed that the main categories of up-regulated genes were carbohydrate metabolism and protein degradation. Responses to reactive oxygen species and hydrogen peroxide metabolism also constituted a large group of functionalities. GO enrichment analysis on down-regulated genes showed that main categories included functionalities involved in transcription, RNA metabolism and translation. Results of full GO term analysis are recorded in the Supplementary file 3. Analysis of transcriptomes showed that 2026 genes increased their transcript levels at breaking of dormancy in both (T0-T1 and T0-T1SA) conditions. 398 genes had increased transcript levels in the control condition and 888 genes had increased levels in the sorbic acid condition. Comparison of GO enrichment results of up-regulated genes revealed that categories such as GO:0008652 (cellular amino acid biosynthetic process), GO:0009165 (nucleotide biosynthetic process), GO:0046467 (membrane lipid biosynthetic process), GO:0033108 (mitochondrial respiratory chain complex assembly) and GO:0032543 (mitochondrial translation) were absent in the sorbic acid data set. Analysis of down-regulated genes during the first hour of germination showed that 1946 genes decreased their transcript level in both conditions (T0-T1 and T0-T1SA). 404 genes had decreased transcript levels in the non-sorbic acid condition and 781 genes had decreased transcript levels in the sorbic acid condition. GO terms related to oxidative phosphorylation (mitochondrial electron transport/ubiquinol to cytochrome *c*, GO:0006122, and mitochondrial respiratory chain complex assembly, GO:0033108) were not present in the sorbic acid data set (Supplementary files 3 and 5). Equally, transcription of proton pumps was highly up-regulated in the control condition but changes in sorbic acid media were smaller.

In order to examine cellular responses to sorbic acid exposure at the protein level, we performed a qualitative proteomic study of conidia germinated for 1 h in the presence (T1SA) and absence (T1) of sorbic acid. We also explored the protein content at the 5 h time point of conidia germinated in the presence (T5SA) of sorbic acid to detect the delayed changes in expression as a consequence of sorbic acid and compared the data with the non-sorbic acid data set (T5). There were 535 proteins detected in T1SA and 89 of them were T1SA-specific and were not found in the T1 proteome. There were 759 proteins detected in T5SA and 319 of them were T5SA-specific and not detected in the T5 control condition. A list of all the reported proteins is provided in Supplementary file 2. Individual proteins are discussed below in context with the transcriptome data.

**Fig. 4:**
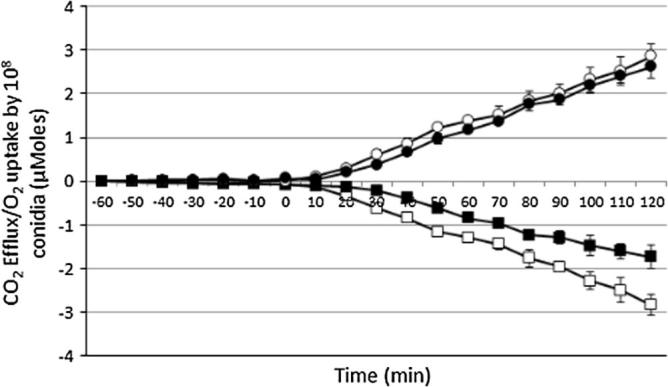
O_2_ uptake (open squares) and CO_2_ efflux (open circles) by 10^8^ conidia without and with sorbic acid (closed squares, closed circles) over the period of 120 min of germination at 28 °C. The means and standard deviations of duplicate samples are shown. ACM ± sorbic acid was added into conidia resuspended in water at time 0.

We showed that germinating conidia fully switch to respiration, following a period of fermentation, after triggering of outgrowth. However, when sorbic acid was present in the germination medium, the fermentation stage continued for longer (>30 min) after germination triggering (Fig. 2A). The impact of sorbic acid on CO_2_ efflux was negligible, however, with O_2_ uptake being much slower and therefore respiration was delayed (Fig. 4). In order to explore the impact of sorbic acid on nutrient uptake we followed the uptake of radioactively-labelled glucose. Conidia germinated for 5 h in the presence and absence of sorbic acid and samples were taken every 20 min. The results showed (Fig. 3B) that uptake of glucose starts at 60 min after triggering of germination in the control condition, but there was no detectable uptake of glucose in the sorbic acid condition during the first 200 min. The delay in uptake meant that only 90 nmol of glucose were taken up during 5 h by 10^7^ conidia in comparison to 300 nmol taken up in the control condition. Genes encoding putative glucose/hexose transporters had lower transcript levels in the sorbic acid condition than in the control sample, apart from An05g01290. Glucokinase (An12g08610) is a high affinity glucose phosphorylating enzyme necessary for the activation of intracellular glucose and it plays an important role in the germination process especially in low glucose conditions (Fleck and Brock, 2010). The presence of this protein was detected in T5SA. Trehalose was completely catabolised within the first 0.5 h of germination, whereas in sorbic acid trehalose depletion took 1 h (Fig. 3A). Also, the transcript level of *treB* (An01g09290), encoding neutral trehalase, was higher in sorbic acid-treated conidia. The *facB* gene that encodes the main regulator of acetate metabolism was up-regulated by sorbic acid. the transcript level of *facA* (An04g05620) was also higher in sorbic acid and the FacA protein was detected in the T1SA proteome. Genes encoding acetyl-CoA acyltransferases (An16g09190, An02g03320) catalyzing the last step of β-oxidation were also induced by sorbic acid and proteins were present in the sorbic acid proteomes. Transcriptional activation of β-oxidation in *A. nidulans* is regulated by cutinase transcription factors, regulators of fatty acid catabolism (Hynes et al., 2006). The *A. niger* genome also contains homologous genes and transcript levels of An16g08980, An08g09490, An18g03930 and An01g13410 were elevated in the sorbic acid condition (Table 2).

Our transcriptome and proteome data showed that sorbic acid differentially regulated transcription of genes involved in plasma membrane remodeling and relevant proteins were also detected. Metabolism of ergosterol, was also affected by sorbic acid. Transcription of the sterol regulatory element binding protein SREBP encoded by *srbA* (An03g05170) that serves as a sterol-dependent oxygen sensor and sterol homeostasis in filamentous fungi (Willger et al., 2008) was up-regulated by sorbic acid. Sterol 24-C-methyltransferase, encoded by *erg6* in *A. fumigatus* catalyses conversion of lanosterol to eburicol and is involved in synthesis of secondary sterols. One of two putative *erg6* genes (An04g04210) was up-regulated by sorbic acid and its respective protein was detected in the 5 h sorbic acid proteome. *erg25* (An03g06410) encoding C-4 methyl sterol oxidase was also up-regulated by sorbic acid. Other genes involved in sterol metabolism had lower transcript levels in the sorbic acid condition.

## 4 Discussion

Fungal conidia are reproductive structures that are dispersed into the air in order to propagate the fungus. The key factor in a battle against fungal pathogenesis and spoilage is to understand their early development. It has been long believed that dormant conidia are metabolically inactive. While that is possibly true of dry conidia, we have shown for the first time that resting conidia in aqueous suspension are not completely dormant as they exhibit a low level of respiratory metabolism. This result is in contrast to the data of Taubitz et al. (2007) who detected no metabolic activity in suspended conidia of *A. fumigatus*. We calculated, however, that those authors may not have detected the O_2_ consumption over their chosen timescale with the numbers of conidia used (10× fewer than used here). Furthermore, it is possible that higher O_2_ uptake in comparison to CO_2_ efflux in dormant conidia might be associated with O_2_ being used by other metabolic pathways requiring oxygen, e.g. the synthesis of unsaturated fatty acids and sterols. Respiration (O_2_ uptake and CO_2_ efflux) and extra O_2_ uptake was completely inhibited in heat-killed conidia (data not shown). Viability of dormant conidia that came in contact with water decreased in comparison with those that stayed dry (unpublished results), possibly as a result of internal energy sources becoming depleted. Immediate O_2_ uptake, inhibition of respiration by cyanide and the presence of transcripts of electron transport genes suggest that dormant conidia are equipped with a functional electron transport chain. Difference spectrophotometry of the mitochondrial fraction of dormant spores of *Neurospora crassa* showed the presence of all cytochromes of the electron transport pathway (Stade and Brambl, 1981). The inhibitor of alternative oxidase, SHAM, did not have any effect on O_2_ uptake suggesting that alternative respiration is not active in dormancy. Nevertheless, expressed alternative oxidase was detected in dormant *A. niger* conidia but its activity wasn’t measured (Hattori et al., 2008).

As soon as resting conidia detected the availability of an external carbon source, trehalose metabolism was initiated. It was catabolised within 30 min and it correlated with the initial fermentation stage. It is probable that conidia use trehalose as a substrate for fermentation. Mannitol catabolism was also initiated at the breaking of dormancy (Novodvorska et al., 2013). It is not likely, however, that mannitol was metabolised by fermentation, as full respiration was detected at 25 min after initiation of germination but mannitol depletion continued for 2 h. Germinating conidia require a higher rate of respiration in comparison to resting conidia and, in order to achieve this, they need to increase the expression of proteins involved in respiration. It is possible that they take energy for this from fermentation, most likely by metabolism of trehalose. The reason why conidia might ferment trehalose remains an unanswered question but probably relates to fermentation being much simpler than respiration and a process that can be initiated more quickly. Around 20% of all yeast species have been reported to ferment trehalose (Barnett et al., 2000). Addition of SHAM to germinating conidia did not cause a drop in O_2_ uptake suggesting that alternative oxidase doesn’t operate at conidial outgrowth. The presence of cyanide completely stopped oxygen uptake, indicating electron flux via cytochrome *c*.

Uptake of glucose has been studied before (Hayer et al., 2013) in minimal media and it was detected at 75 min after initiation of germination. We detected that in complex media, glucose uptake was initiated at 60 min after triggering which suggests that the presence of other nutrients has an effect on the timing of transport of glucose into the conidia. Particular amino acids also serve as triggers of germination (Hayer et al., 2014). Signalling pathways that coordinate these processes are largely unknown, although a few key molecules have been identified in other *Aspergilli* (Lafon et al., 2005, Brown et al., 2015, De Assis et al., 2015). The GRCR pathway is also well conserved (Mah and Yu, 2006, Yu, 2006, Ogawa et al., 2010) although a G(γ) subunit homolog is not present in the genome of the strain of *A. niger* used here. PtcE phosphatase was discovered to be essential in glucose sensing and a mutant strain is unable to germinate on glucose in *A. nidulans* (De Assis et al., 2015). The homologous protein in *A. niger* was detected in the T1 proteome and there were high transcript levels in dormant conidia suggesting its role in glucose sensing. Hydrophobicity of conidia hindered the protein extraction and detection process which may explain the lower number of proteins detected in comparison with the number of transcripts, and that was most acute with T0 conidia (data not shown).

Inhibition of fungal membrane activities is one proposed underlying mechanism behind the strong growth inhibiting effect caused by sorbic acid (Stratford and Anslow, 1998) and inhibition of active transport was reported (Melin et al., 2008, Stratford et al., 2013). It was shown by *in vitro* studies that sorbic acid interacts with the plasma membrane, specifically with the phospholipid headgroups and partially reaches the hydrophobic core of the lipid bilayer (Chu et al., 2009). In order to maintain the integrity of the plasma membrane, expression of genes associated with ergosterol biosynthesis was adjusted in response to sorbic acid. Our data correlate with findings made in *Penicillium roqueforti* that potassium sorbate alters the composition of phospholipids, neutral lipids and sterols (Sergeeva et al., 2009). Impact of sorbic acid on the plasma membrane was also suggested in *Bacillus subtilis* (Ter Beek et al., 2008). SrbA acts as a transcriptional regulator of sterol biosynthesis in *A. fumigatus* (Willger et al., 2008) and its up-regulated transcription implies distorted ergosterol synthesis/homeostasis of conidia germinating in a sorbic acid environment. Increased expression of Erg6 suggests that *A. niger* modulates the ergosterol pathway by favouring ethyl sterols as a consequence of sorbic acid perturbation of the plasma membrane. The *erg25* gene, catalyzing C4-demethylation, had increased transcript levels implying that this may be a limiting step of ergosterol biosynthesis in the presence of sorbic acid. Possible inhibition of oxygen flux through the plasma and mitochondrial membranes by sorbic acid may have delayed the onset of respiration allowing continued fermentation. This hypothesis is also supported by lower expression levels of proton pumps in comparison to the control condition. Also, some GO terms related to respiration were missing from the up-regulated set of genes (T0-T1SA). We have unpublished evidence that sorbic acid distorts production of cellular energy (ATP) in germinating conidia and that may be the consequence of prolonged fermentation and delayed onset of respiration. Glucokinase is an enzyme necessary for the activation of intracellular glucose. Increased transcription of the gene encoding glucokinase and the presence of protein in the T5SA proteome probably indicates a response to lowered levels of glucose inside the cell most likely caused by deficient glucose uptake and/or trehalose catabolism.

We report for the first time that dormant conidia suspended in an aqueous environment are not metabolically inactive as they exhibit respiratory metabolism. Spore germination initiated fermentation and was quickly replaced by respiratory metabolism. Additionally, sorbic acid, as a membrane-active compound, restricted the uptake of glucose and delayedg the catabolism of trehalose. Also, the fermentation phase was prolonged and initiation of respiration was delayed.

## Figures and Tables

**Table 1:** Selection of genes with changed transcript levels at T1 (1 h after germination) in comparison to T0 (dormant conidia). RPKM is defined in Section 2.

Gene	T0 RPKM	T1 RPKM	Verified/putative function
An11g04810	3.1	94.8	Alternative oxidase, *aox1*
An01g09290	85.1	17.6	Neutral trehalase, *treB*^a^
An02g03540	15.8	489.9	Low affinity glucose transporter, *mstE*
An15g03940	4.0	336.7	Monosaccharide transporter
An05g01290	2.8	324.2	Monosaccharide transporter
An16g08940	5.1	118.8	Monosaccharide transporter
An02g01270	1.6	25.8	Monosaccharide transporter
An12g07450	1.6	9.5	High affinity sugar/H + symporter, *mstA*
An02g01560	0.1	1.4	GPCR
An09g06510	6.1	2.8	GPCR
An08g04110	1.4	4.8	GPCR
An07g09280	4.7	17.4	GPCR
An01g00400	0.8	1.2	GPCR
An08g06130	8.6	89.2	GTP-binding protein α subunit, *fadA*
An02g08000	5.2	3.3	GTP-binding protein α subunit, *ganA*
An18g02090	12.0	35.4	GTP-binding protei β subunit, *sfaD*
An02g03160	2.2	47.9	*flbA,* RGS protein
An18g06110	2.6	11.4	*rgsA,* RGS protein
An01g03700	2.5	17.7	Regulating carbon source dependent germination, *psrA*
An01g14160	4.9	21.5	Regulating carbon source dependent germination, *ptcD*
An02g06630	2.0	6.3	Regulating carbon source dependent germination, *ptpB*
An07g07110	6.2	4.7	Regulating carbon source dependent germination, *mgsA*
An18g05890	12.7	2.1	Regulating carbon source dependent germination, *ptcE*^a^

a Protein detected in T1 proteome.

**Table 2:** Selection of genes with changed transcript levels as a consequence of sorbic acid presence (T1SA) in comparison with non-sorbic acid condition (T1) and presence of associated protein. RPKM is defined in Section 2.

Gene	T1 RPKM	T1SA RPKM	Putative/verified function lipid/fatty acid catabolism	Presence of protein
An16g09190	145.5	102.2	Acetyl-CoA C-acyltransferase	T1SA
An02g03320	20.5	53.0	Acetyl-CoA C-acyltransferase	T5SA
An01g12960	7.7	11.8	Butyryl-CoA dehydrogenase	T5SA
An03g03360	16.8	15.3	Carnitine/acyl carnitine carrier, *acuH*	T5SA
An16g08980	5.7	104.9	Cutinase transcription factor, *farB*	
An08g09490	2.4	87.0	Cutinase transcription factor	
An18g03930	26.9	50.9	Cutinase transcription factor	
An01g13410	9.9	43.2	Cutinase transcription factor	
An16g07110	39.6	242.5	Acetyl-CoA hydrolase	T1, T1SA, T5SA
An08g06580	42.3	145.5	Acetate regulatory protein *facB*	
An04g05620	17.2	23.2	Acetyl-CoA synthase, *facA*	T1SA
An16g05340	5.3	8.1	Enoyl reductase	T5SA

			Plasma membrane remodeling	
An09g01240	12.6	80.8	Lysophospholipase	T1SA
An04g03830	0.5	2.0	GPI-anchored cell wall protein	T5SA
An01g00050	197.3	85.9	Fatty acid metabolism	T1, T1SA, T5SA
An04g04210	6.4	862.7	δ-24-sterol-C-methyltransferase, *erg6*	T5SA
An03g06410	1.8	10.4	C-4 methyl sterol oxidase, *erg25*	
An03g05170	31.2	109.9	SREBP, *srbA*	
