# Supplementary material for: Metabolic activity in dormant conidia of Aspergillus niger and developmental changes during conidial outgrowth
Source: Fungal Genet Biol. 2016 Sep;94:23–31. doi: 10.1016/j.fgb.2016.07.002 (PMC4981222; doi:10.1016/j.fgb.2016.07.002)

T0-T1 up-regulated (trancriptome)

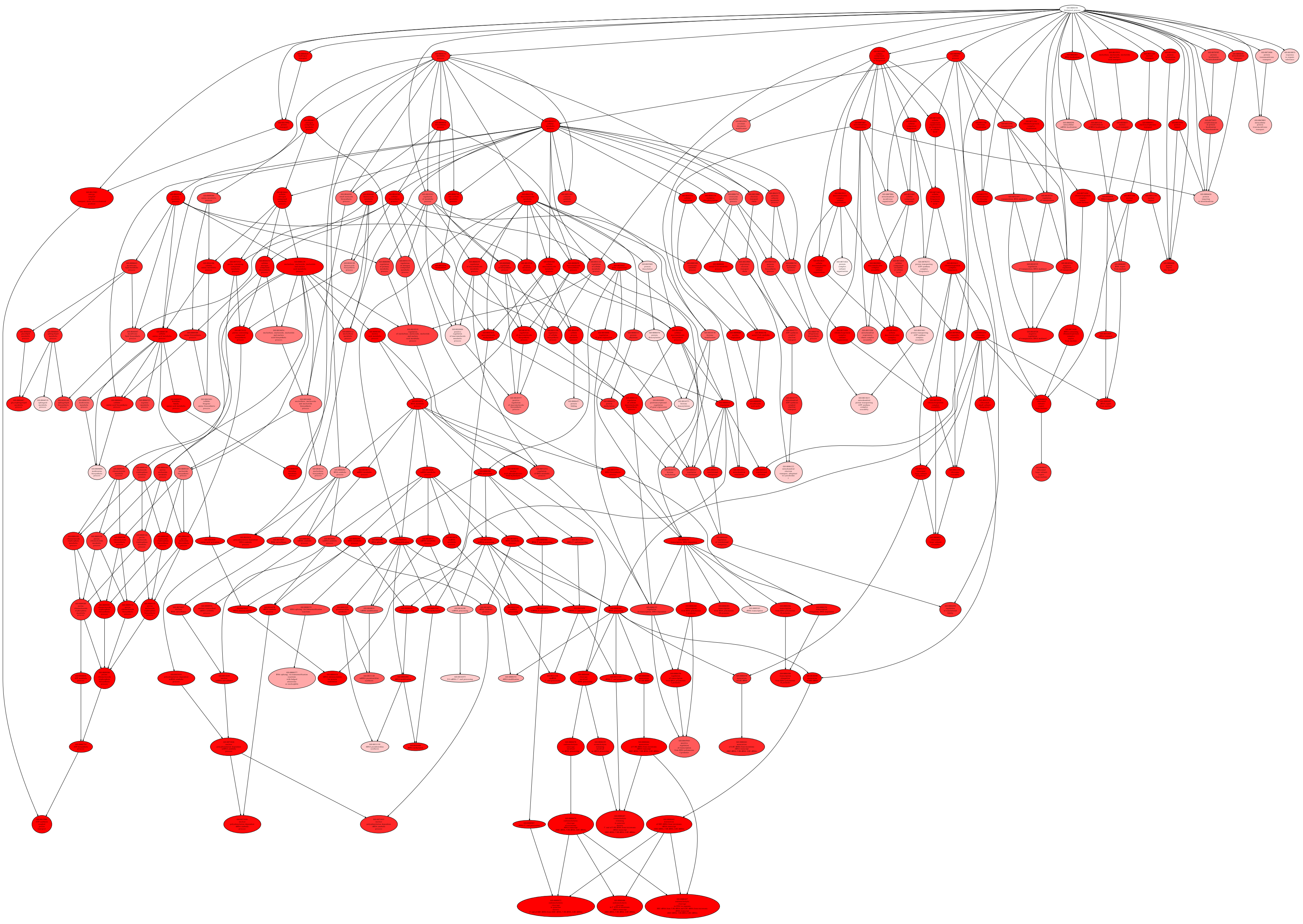

T0-T1 down-regulated (trancriptome)

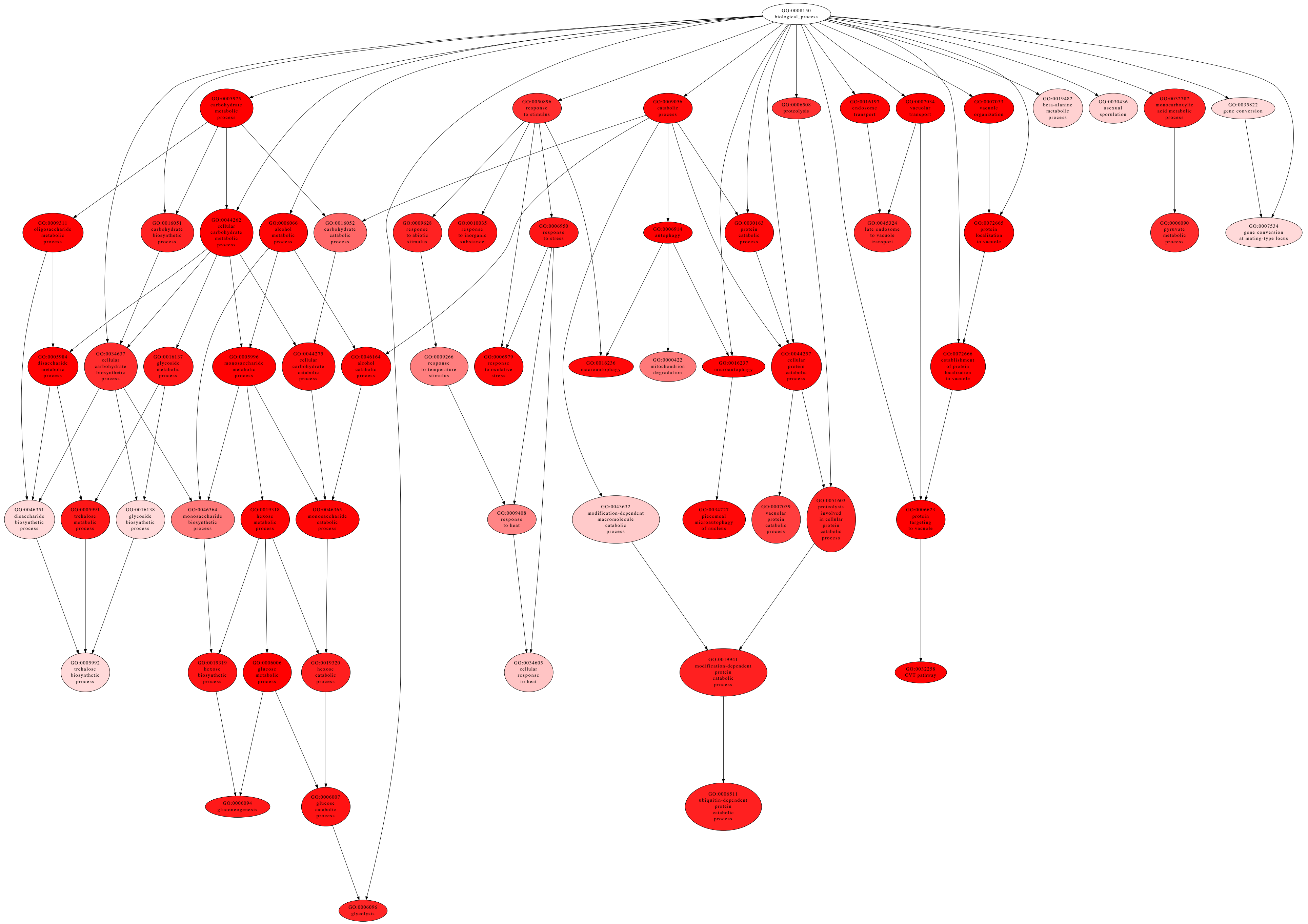

T0-T1SA up-regulated (trancriptome)

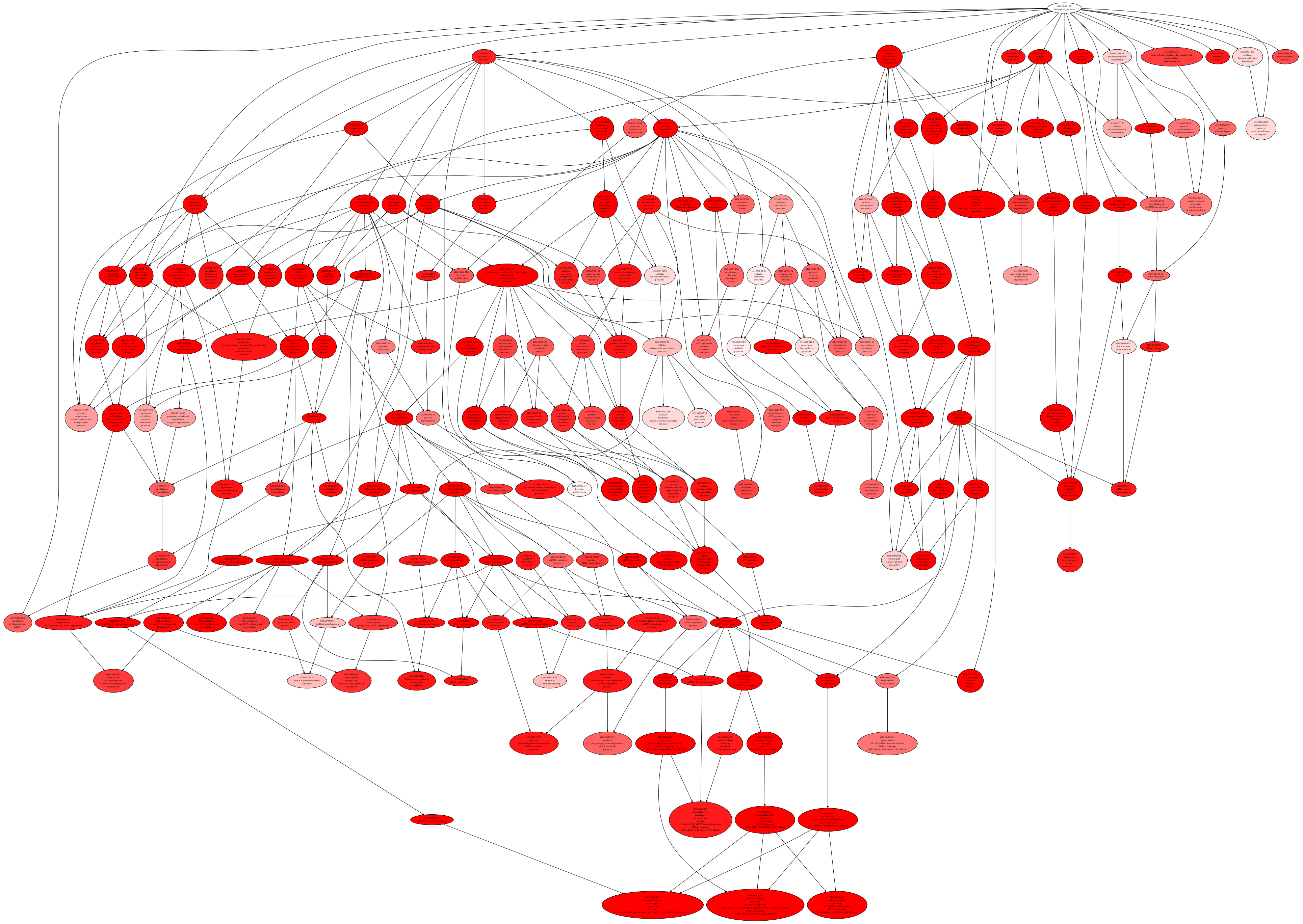

T0-T1SA down-regulated (trancriptome)

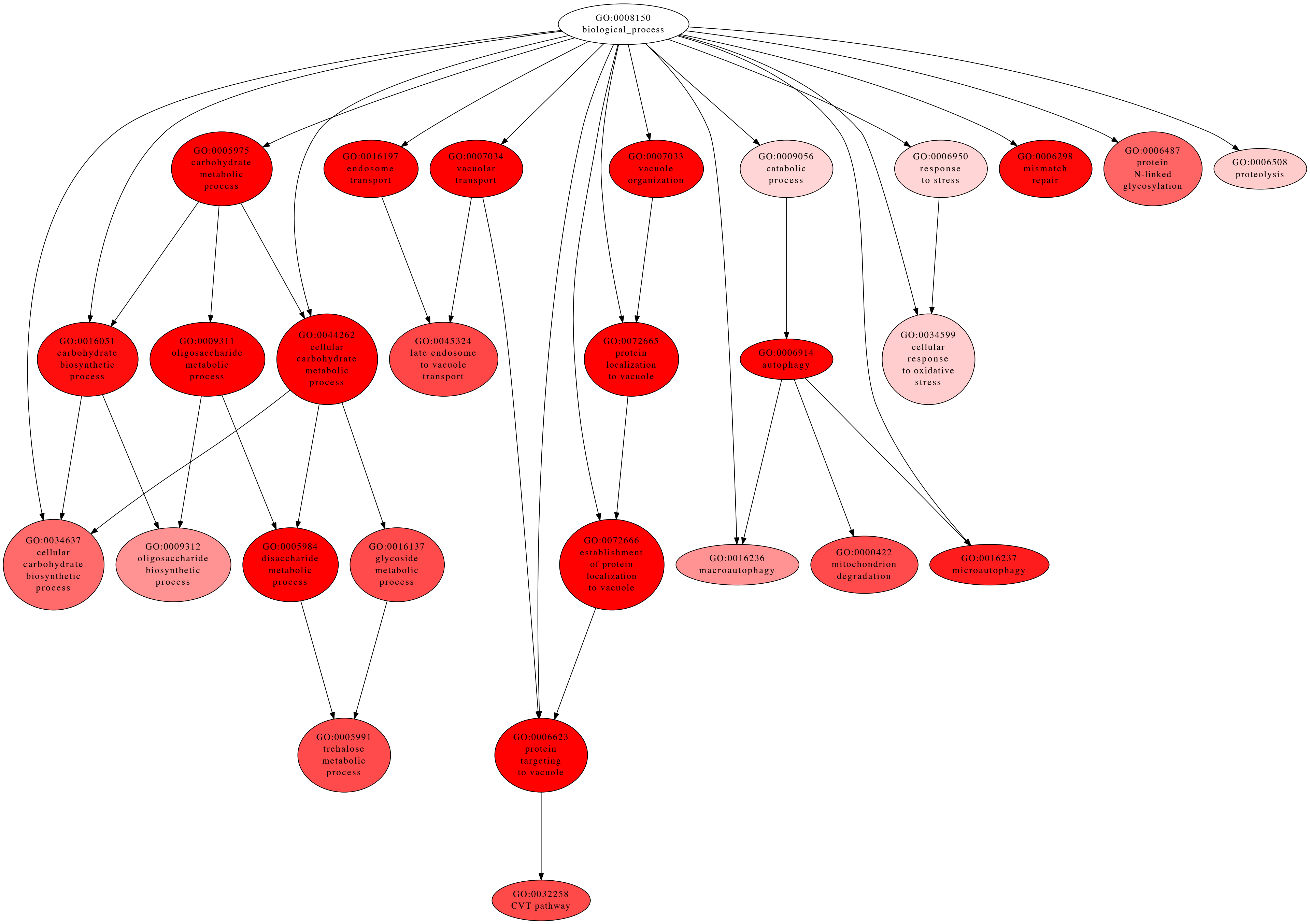

T1-T1SA up-regulated (trancriptome)

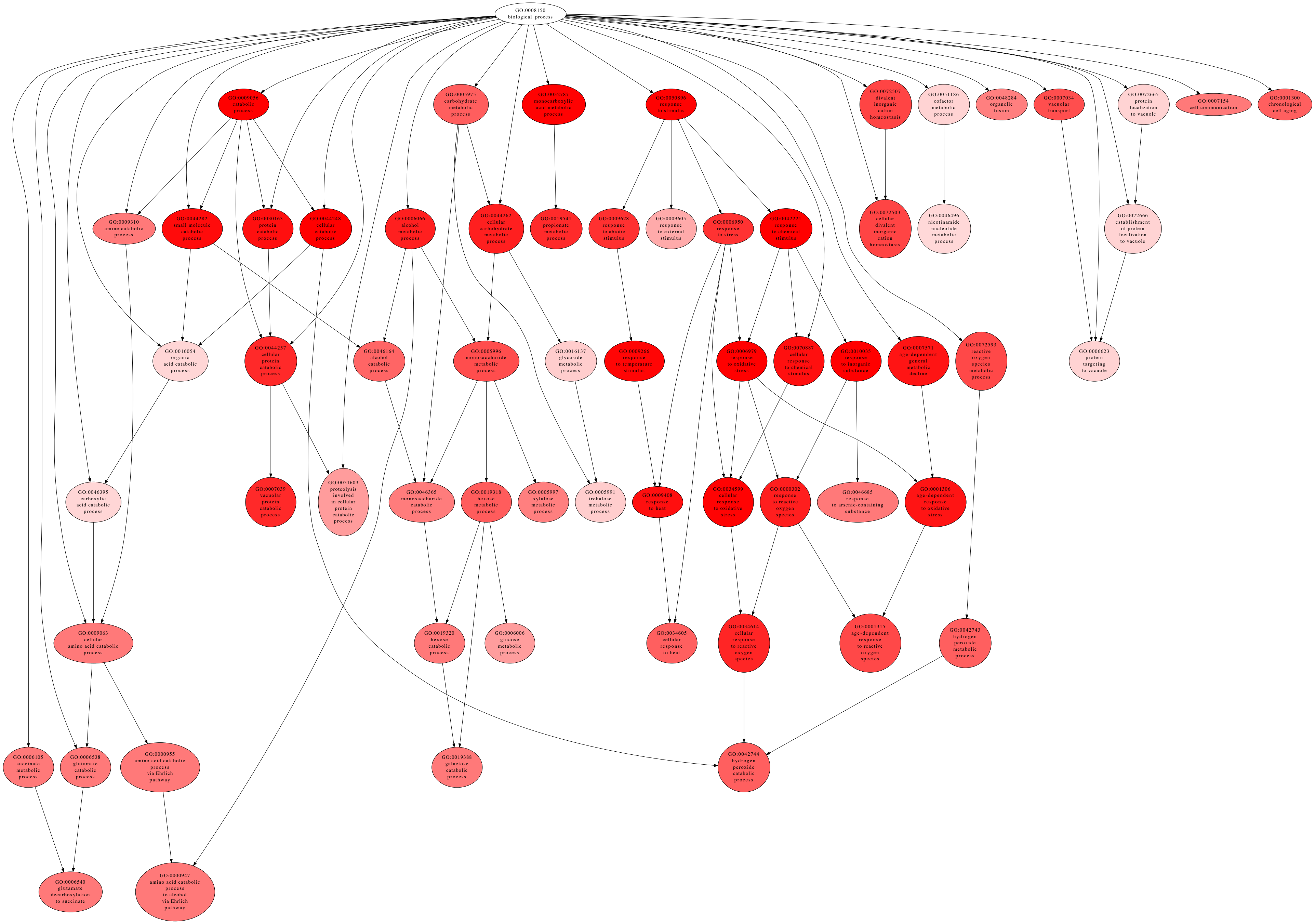

T1-T1SA down-regulated (trancriptome)

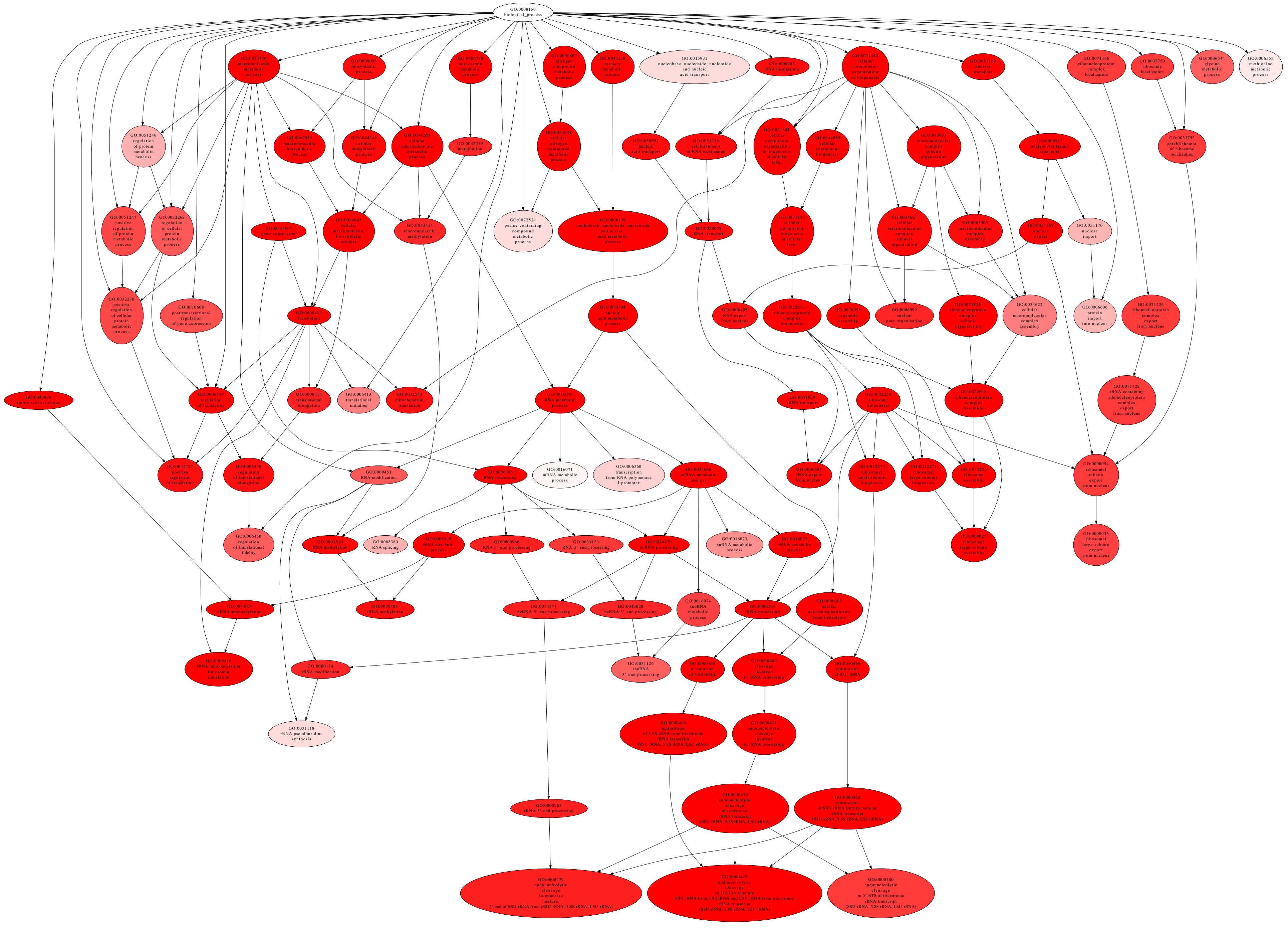

T1 (proteome)

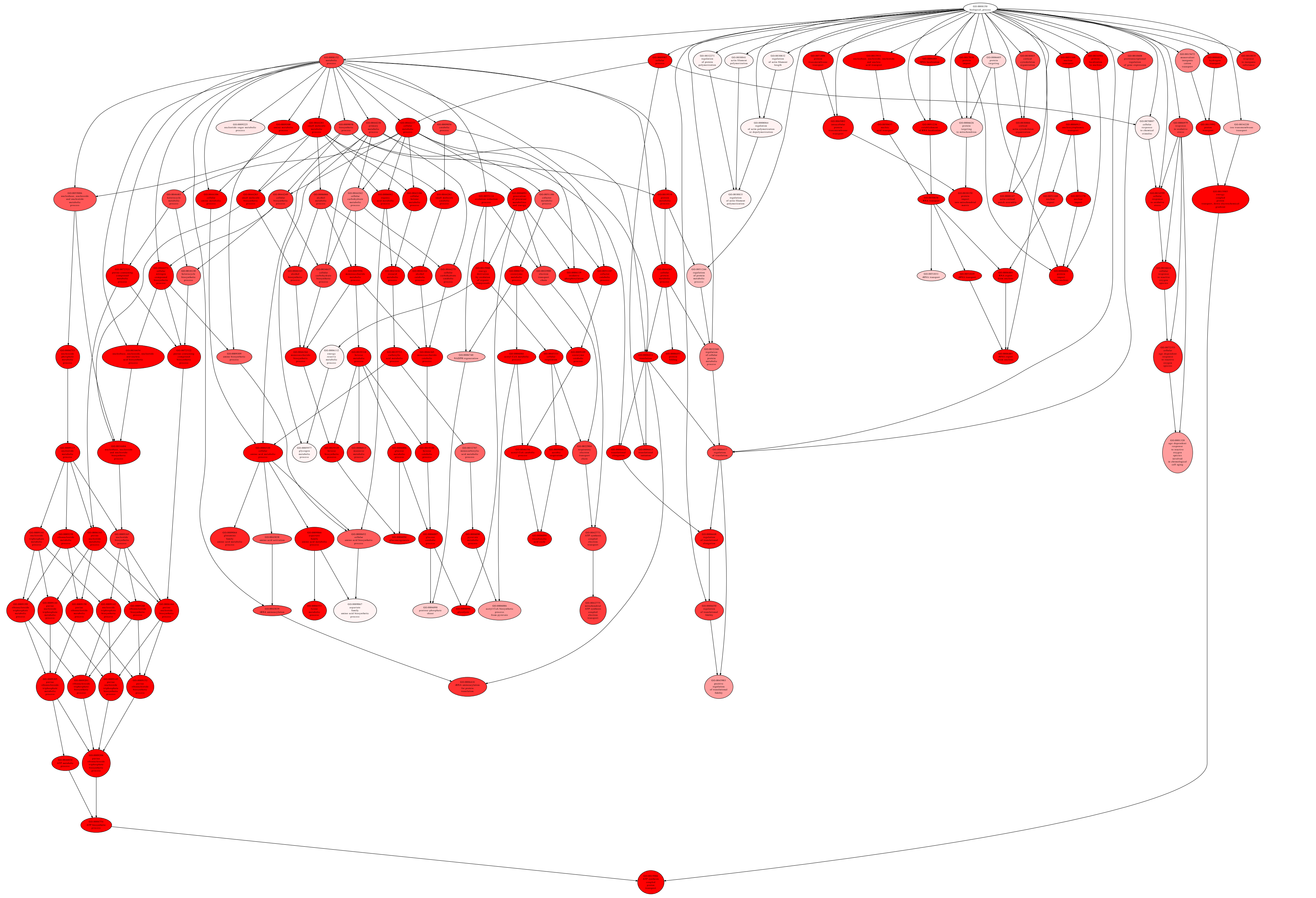

T1SA (proteome)

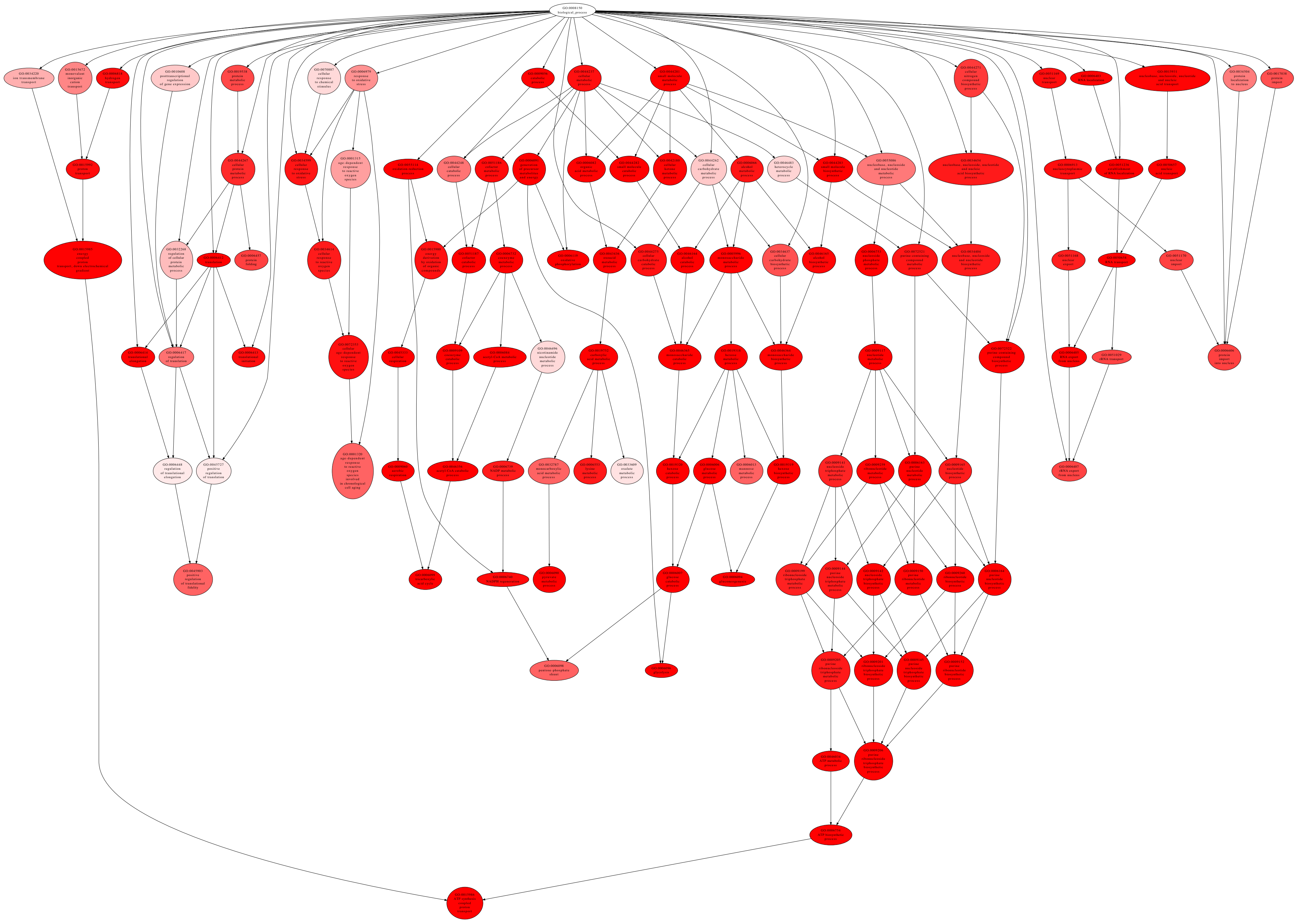

T5 (proteome)

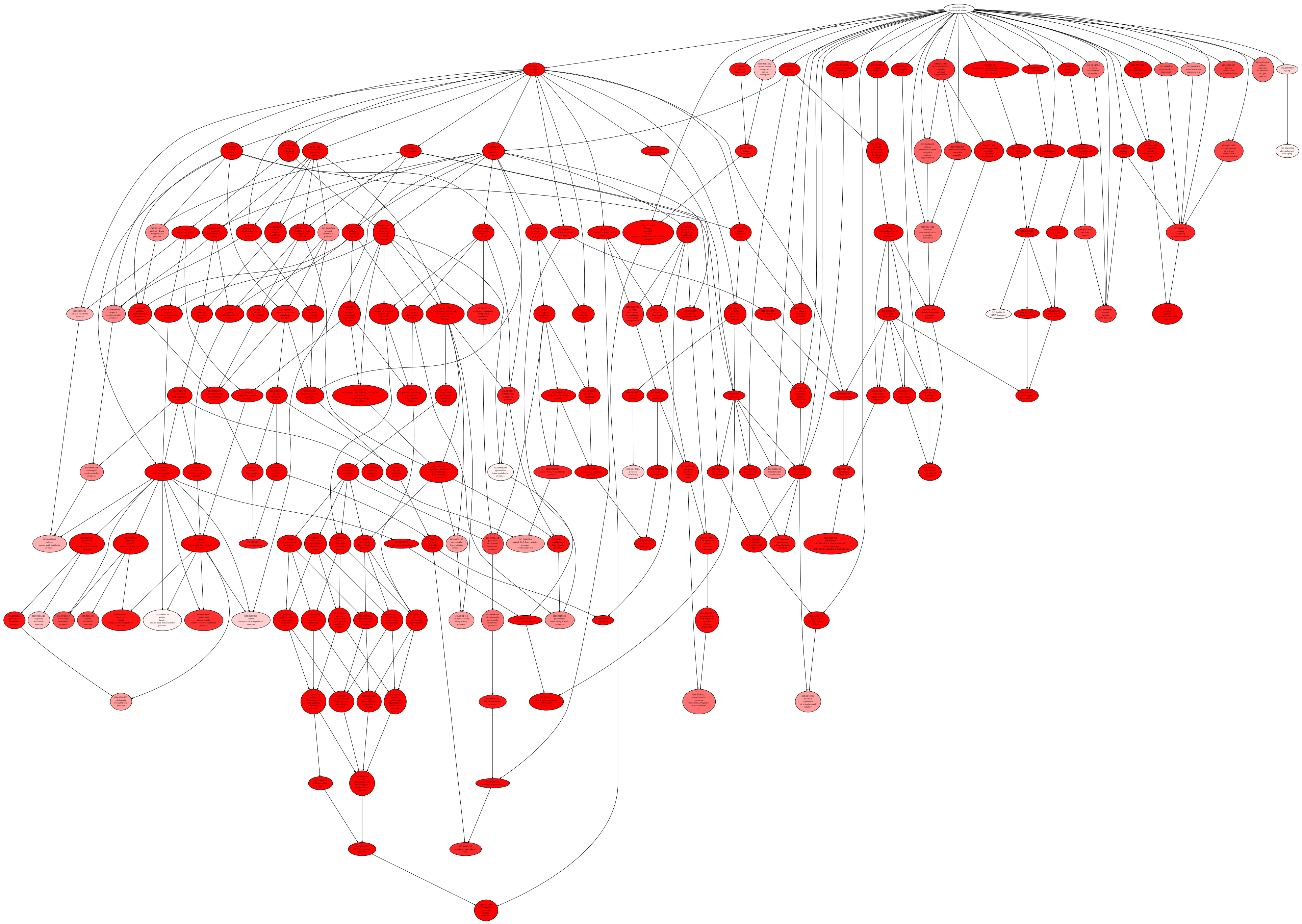

T5SA (proteome)

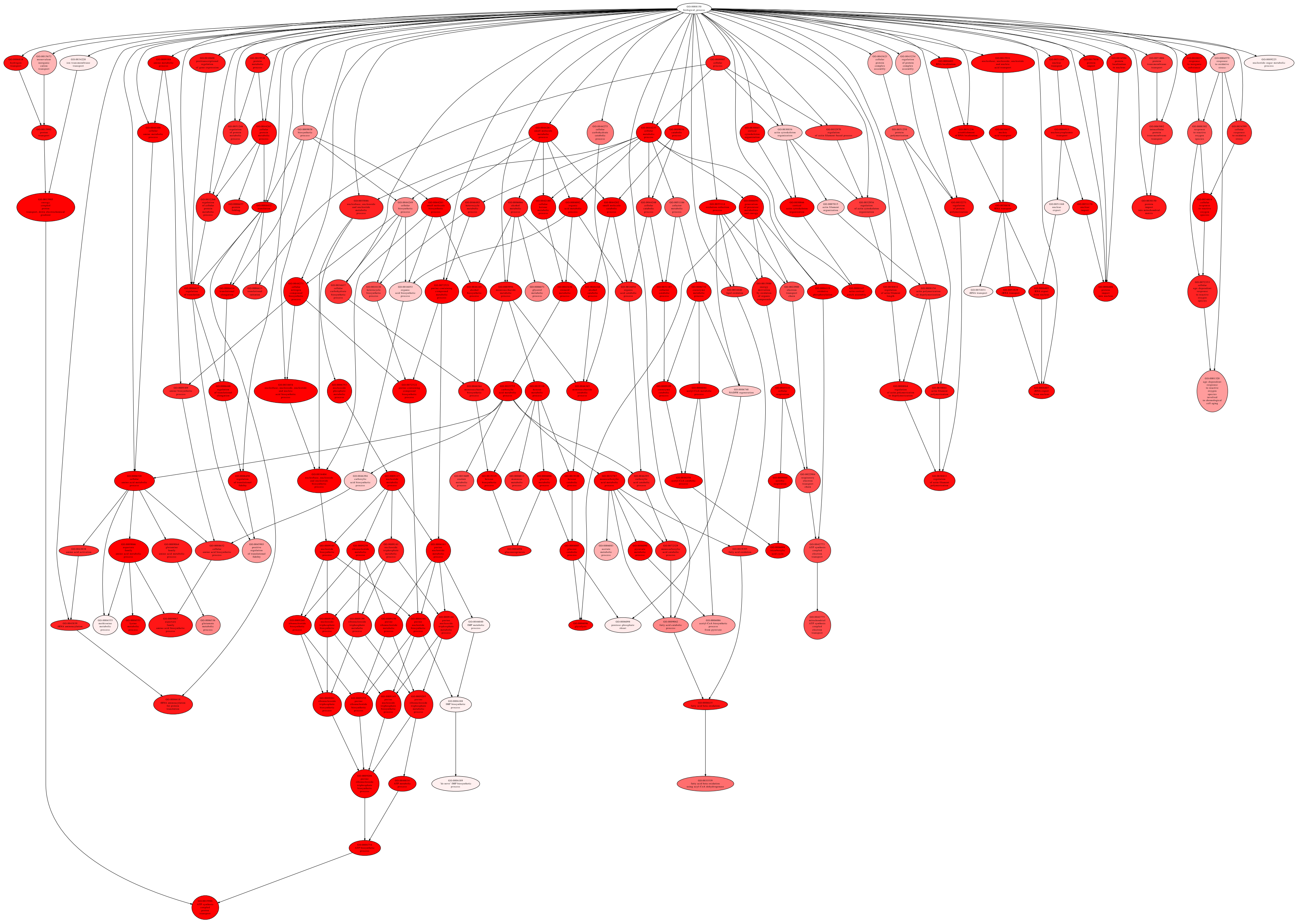

Supplement: Supplementary data 5 [file mmc5.pdf]
